# Time, Trust and Participation: A Nationwide Quantitative Study of Person‐Centred Care in Forensic Mental Health Settings

**DOI:** 10.1111/inm.70383

**Published:** 2026-10-27

**Authors:** Valentina Vidaurrázaga Aras, Axel Wolf, Thomas Nilsson, Eirini Alexiou, Sepideh Olausson

**Affiliations:** ^1^ Institute of Health and Care Sciences, Sahlgrenska Academy University of Gothenburg Gothenburg Sweden; ^2^ Department of Forensic Psychiatry Sahlgrenska University Hospital Gothenburg Sweden; ^3^ Center for Ethics, Law, and Mental Health (CELAM), Sahlgrenska Academy University of Gothenburg Gothenburg Sweden; ^4^ Department of Anesthesiology and Intensive Care Sahlgrenska University Hospital Gothenburg Sweden; ^5^ Institute of Nursing and Health Promotion Oslo Metropolitan University Oslo Norway

**Keywords:** forensic psychiatry, patient participation, person‐centred care, quality of health care, questionnaire

## Abstract

Person‐centred care emphasises the uniqueness of individuals through partnerships, the co‐creation of care plans and coordinated health care. Its implementation in forensic mental health services is challenged by legal coercion and restrictive practices. Person‐centred principles are embedded in legislation; however, national data on patients' perspectives on person‐centred care are lacking. This study aimed to map person‐centred care as perceived by patients and to identify areas for improvement. A nationwide survey included 329 patients across 13 forensic psychiatric hospitals in Sweden using the Generic Person‐Centred Care Questionnaire supplemented with contextual background questions. The median Generic Person‐Centred Care Questionnaire score was 70 (range 19**–**95). Length of stay was associated with perceived person‐centred care; patients hospitalised for 2–5 years reported higher total scores than those hospitalised for more than 10 years, although the pairwise difference did not remain significant after correction for multiple comparisons. Trust in staff differed by length of stay, with patients hospitalised for > 10 years reporting significantly lower trust than those hospitalised for 0–1 and 2–5 years. Communicative safety showed a similar pattern, although pairwise differences were not significant after correction. No significant differences in total scores were found by gender, age, ethnicity, diagnosis, or hospital type. Overall, patients reported positive experiences of respect and emotional support. Among those who reported having a written care plan, involvement in and understanding of the plan were rated positively, whereas broader involvement in decision‐making, use of patients' own resources and relatives' participation received lower ratings, indicating areas for further improvement in person‐centred practice.

## 1 Introduction

Forensic mental health services (FMHS) operate at the intersection of health care, criminal justice and public safety. Internationally, patients in FMHS often present with severe mental illness and complex psychosocial challenges and are typically admitted to care against their will (Leclair et al. 2026). International trends indicate increasing lengths of stay, growing demand for secure hospital capacity, and increasingly complex patient care needs within FMHS. These developments have raised concerns regarding the effectiveness and sustainability of existing care models, highlighting the need for interventions that strengthen recovery, rehabilitation and person‐centred practice (Dima et al. 2024; Leclair et al. 2026; Tomlin et al. 2021). Person‐centred care (PCC) is an ethical and practical framework that seeks to establish partnerships between patients and healthcare professionals by recognising patients' experiences, values and preferences as central to care. Across multiple healthcare settings, PCC has been associated with increased patient satisfaction, enhanced self‐efficacy and improved symptom management (Britten et al. 2020; European Committee for Standardization 2020; Wolf et al. 2024).

However, the operationalisation of PCC within restrictive and coercive care environments remains underexplored and insufficiently evaluated.

### 1.1 Background

Patients within FMHS are cared for under legal mandates, subjected to stringent security measures, and cared for within institutions that prioritise safety and societal protection over autonomy (Beis et al. 2022). In Sweden, FMHS are structured into high‐, medium‐ and low‐security levels, with a median length of stay of 7 years, which is considerably longer than that in general psychiatry, reflecting the complexity of the system and patient conditions treated (Sivak et al. 2023). This study focused on low‐ and medium‐security wards, referred to as rehabilitation wards, as they emphasise therapeutic interventions and recovery frameworks (Svennerlind et al. 2010). Furthermore, national reports in Sweden have emphasised the urgent need for evidence‐based interventions that are clinically effective and informed by reflexive ethical reasoning, supporting continuity, participation and therapeutic alliances in FMHS (Swedish Agency for Health Technology Assessment and Assessment of Social Services (SBU) 2018).

In clinical practice, PCC represents a shift from professionally dominated models of care toward relational and dialogical approaches that seek to balance power, foster trust, and support active patient involvement through shared decision‐making, co‐created care plans and coordinated care (Ekman et al. 2011; McCormack and McCance 2006). However, within highly structured and security‐oriented environments, opportunities for autonomy and meaningful participation may be restricted, contributing to patients' experiences of powerlessness and reduced agency (El‐Alti et al. 2022; El‐Alti 2023). Many individuals in FMHS have histories of complex trauma, underscoring the importance of relational safety, trust and stability to enable engagement in treatment (Marklund et al. 2020; Meddeb et al. 2022; Simjouw et al. 2024). Staff's dual responsibilities between therapeutic goals and legal obligations reflect broader tensions between recovery‐oriented ambitions and security‐driven practices, potentially undermining the realisation of PCC principles (El‐Alti et al. 2022; El‐Alti 2023; Merkt et al. 2021; Söderberg et al. 2020).

Nevertheless, emerging evidence suggests that PCC principles can be successfully adapted to FMHS, with potential benefits for participation, motivation and recovery (Hauso et al. 2021; Holley et al. 2020). Implementation studies in FMHS have demonstrated improvements in shared decision‐making and participation in care planning over time (Hauso et al. 2021; Livingston et al. 2013, 2012). Similarly, Holley et al. (2020) emphasised that practical PCC approaches, such as fostering meaningful engagement, providing continuity of care and building partnerships, were pivotal in supporting motivation and progress in forensic psychiatric rehabilitation.

However, recent reviews conclude that empirical evidence remains limited and few implementation studies of collaborative models emphasising PCC principles have been conducted in FMHS. Most contemporary research relies on qualitative designs, single‐site interventions, or evaluations of shared assessment, limiting generalisability and knowledge of how these approaches can be embedded in routine FMHS practice (Luigi et al. 2025; Zhao et al. 2025). Quantitative assessments from the patient perspective are particularly scarce.

To our knowledge, no nationwide study has assessed the perceptions of PCC among individuals receiving forensic psychiatric care. Addressing this gap is essential given the prolonged lengths of stay and ethically complex nature of FMHS. Hence, this study aimed to investigate the following research questions:

1. What is the current state of PCC as perceived by patients in Swedish forensic psychiatric rehabilitation units?
2. Do patients' perceptions of PCC differ according to background characteristics?
3. Which key areas for improvement can support the contextually appropriate implementation of PCC in FMHS?

## 2 Methods

### 2.1 Design and Instruments

A quantitative design using the Generic Person‐Centred Care Questionnaire (GPCCQ) and context‐specific background questions was used. The GPCCQ instrument is designed to measure PCC from the patient's perspective across diagnoses and healthcare settings and has been tested in diverse care environments, including psychiatry. Its development and validation included qualitative interviews and Rasch analysis. Internal consistency was evaluated using the Person Separation Index, which was 0.84 (Fridberg et al. 2020).

The GPCCQ includes 20 items assessing PCC as an overarching dimension. Nineteen items are rated on a 5‐point Likert scale, ranging from 1 (‘No, not at all’) to 5 (‘Yes, completely’), with an additional ‘Not applicable’ option. Item 18 assesses whether the patient and staff have developed a written care plan (Yes/No/Not applicable) and serves as a filter item. Respondents answering ‘Yes’ were directed to two follow‐up items (Q18a–b) assessing their involvement in and understanding of the care plan.

In addition to the GPCCQ items, the survey included questions on six background characteristics (gender, length of stay, age, ethnicity, diagnosis and hospital type) and five contextual background questions. The contextual background questions used a three‐point categorical response scale and were designed to contextualise responses from patients receiving care in FMHS. These items aimed to capture the core aspects of a secure and coercive care environment, including the willingness to participate, perceptions of safety and security, trust and communicative safety. To assess the feasibility and comprehensibility of the survey, a pilot test was conducted with four patients, who completed the questionnaire and provided feedback on its content and relevance. Two participants indicated that items concerning written care plans or involvement of relatives were not applicable to their current circumstances. Specifically, they found these questions confusing because they were unaware of the existence of a written care plan or had no ongoing contact with relatives. Overall, the survey was reported to be clear and contextually appropriate. This feedback was interpreted as reflecting limitations in the care context rather than deficiencies in the questionnaire. These patients were not involved in the study design or in the subsequent data analysis.

### 2.2 Procedure

Data were collected between February 2022 and February 2024. All forensic mental healthcare providers (*n* = 25) in Sweden were invited via email to participate in this study. Reminders were sent throughout the data collection period. Following initial contact, written information and consent forms were sent to the Chief Medical Officer (CMO), and each hospital followed its own approval process for participation. In some cases, the first author (VAV) presented the study at management meetings; in others, approval was granted following review by local research councils or directly by the CMO. After institutional approval, a contact person was designated to collaborate with the first author and coordinate survey distribution. Based on the reported numbers of eligible patients, paper surveys were distributed to the wards together with written instructions outlining inclusion and exclusion criteria. The study received ethical approval from the relevant national ethics review authority [details from the Swedish Ethical Review Authority]. Patients received written information describing the purpose of the study, the voluntary nature of participation, confidentiality procedures, and their right to decline or withdraw before returning the anonymous questionnaire, without consequences for their care or legal status. Because questionnaires were anonymous and contained no personal identifiers, individual responses could not be withdrawn after submission. A hospital code was used solely to monitor the number of distributed and returned questionnaires and did not allow identification of individual participants. The return of the completed survey was considered to constitute informed consent. As a token of appreciation, the patients were offered either a scratch lottery ticket or a pack of chewing gum, depending on the hospital's preference. The survey distribution was a multi‐step process involving various personnel before being delivered to the patients.

### 2.3 Sample and Participants

In Sweden, forensic psychiatric care is organised across 25 different providers within 19 regions; providers do not necessarily have their own forensic psychiatric hospitals. Some instead purchase care from specialised secure settings. Inpatient forensic mental health care is delivered across 19 hospitals nationally, of which 13 agreed to participate in this study. Four of the five regional forensic psychiatric hospitals participated, accounting for 51% of the total sample. The participating hospitals were geographically distributed across the country and represented a mix of urban, semi‐urban and rural contexts. This variation supports the representativeness of the sample in relation to the broader structure and delivery of forensic psychiatric services in Sweden. Patients receiving care at security levels 2 and 3 (medium and low security) were eligible for participation. Patients who were unable to understand the study information or provide informed consent were excluded. Eligibility was assessed by ward staff in consultation with the responsible physician according to guidance provided by the first author. Acute psychosis was provided as an example of a clinical condition that could impair the capacity to participate. A total of 783 surveys were distributed across participating hospitals, of which 336 were returned. Of these, 2% were excluded because fewer than 20% of the GPCCQ items had been completed. The final analytic sample therefore consisted of 329 participants (response rate 42%). Given the nationwide design and inclusion of eligible patients within participating hospitals during the data collection period, the sample reflects the accessible population rather than a priori power‐based sampling. The background characteristics of the sample (Table 1) were consistent with national data reported in the Swedish National Forensic Psychiatric Register (Swedish National Forensic Psychiatric Register 2025).

**TABLE 1:** Background characteristics of the study sample.

Background variable	*N* (%)^a^
Total	329 (100)
Gender	
Male	275 (84)
Female	44 (13)
Non‐binary	3 (1)
Missing data	7 (2)
Age	
18–29 years	84 (26)
30–45 years	157 (48)
46–65 years	80 (24)
> 65 years	5 (2)
Missing data	3 (1)
Ethnic background	
Caucasian	241 (73)
Non‐Caucasian	79 (24)
Missing data	9 (3)
Length of stay in forensic psychiatric care	
0–1 year	73 (22)
2–5 years	135 (41)
6–10 years	52 (16)
> 10 years	64 (20)
Missing data	5 (2)
Diagnosis (multiple choices)	
Psychosis spectrum	179 (54)
Neurodevelopmental disorders	61 (19)
Substance abuse disorder	5 (2)
Personality disorder	3 (1)
Depression and/or manic disorder	10 (3)
Do not know/prefer not to respond	59 (18)
Reported two or more diagnoses	120 (37)
Missing data	10 (3)
Hospital type	
Regional	166 (51)
Non‐regional	159 (48)
Missing data	4 (1)

^a^ Percentages may not total 100% due to rounding.

### 2.4 Data and Statistical Analyses

The analysis was structured around the three objectives of this study.

To address objective 1 (mapping the current state of PCC), total scores were calculated from 19 Likert‐scale items. In accordance with the filter procedure described by Fridberg et al. (2020), Q18 was excluded from the total score, while Q18a–b were retained only when Q18 was answered ‘*Yes*’; otherwise, they were treated as missing. ‘Not applicable’ responses were also treated as missing. For participants with at least 16 valid items, total scores were prorated by multiplying the mean of the available items by 19, resulting in a possible range of 19–95. Higher scores indicate greater perceived PCC. Total scores were grouped into low, medium and high categories using the 25th and 75th percentiles as cut‐offs.

Descriptive statistics were calculated for item‐level GPCCQ responses and contextual background questions. For the GPCCQ items, responses of 4–5 were categorised as positive, 3 as neutral and 1–2 as negative. Percentages were calculated from valid responses. Item‐level means and standard deviations were calculated using the original 1–5 response scale. Item 18 was reported as a categorical filter item (*Yes/No*) and Q18a–b were included in item‐level analyses only for respondents who answered ‘*Yes*’ to Q18. Consequently, the analytic *n* varied between items.

To address objective 2 (examining whether patients' ratings of PCC differed across background characteristics), inferential statistics were applied. Group differences in GPCCQ total scores were analysed using Welch's independent‐samples *t*‐test for background characteristics with two groups and Welch's one‐way ANOVA for variables with more than two groups. Although gender subgroup sizes were imbalanced, the analysis was retained while acknowledging limited statistical power. Chi‐square tests were conducted to examine associations between GPCCQ total score categories (low, medium, high) and background characteristics. In addition, all five contextual background questions (BQ1–BQ5) were examined across the background characteristics. Responses were dichotomised as ‘Yes’ (1) versus ‘No/Partly’ (0); thus, mean values represent the proportion responding ‘Yes’. Group differences were analysed using Welch's *t*‐test or Welch's ANOVA, as appropriate. Where significant overall differences were identified for variables with more than two groups, Games–Howell post hoc comparisons were performed with Bonferroni–Holm correction for multiple comparisons (Aickin and Gensler 1996).

To address objective 3 (identifying key areas for improvement), the mean scores for individual GPCCQ items were examined to identify relative strengths and areas for improvement in PCC delivery. Item‐level analyses were used to identify specific dimensions of PCC that may be masked by the overall total score, thereby enabling a more clinically meaningful interpretation of areas that may require targeted improvement. In addition, descriptive analyses of the contextual background questions were used to further contextualise the findings and identify setting‐specific areas for improvement in FMHS. All analyses were performed using the IBM SPSS Statistics software (IBM Corp. 2021).

## 3 Results

A total of 329 patients from 13 forensic psychiatric hospitals with medium and low security wards participated in this study.

### 3.1 Mapping Patients' Perception of Person‐Centred Care

Total GPCCQ scores ranged from 19 to 95, with a median score of 70 (IQR 55.9–85.0), indicating variation in perceived PCC (Figure 1).

**FIGURE 1:**
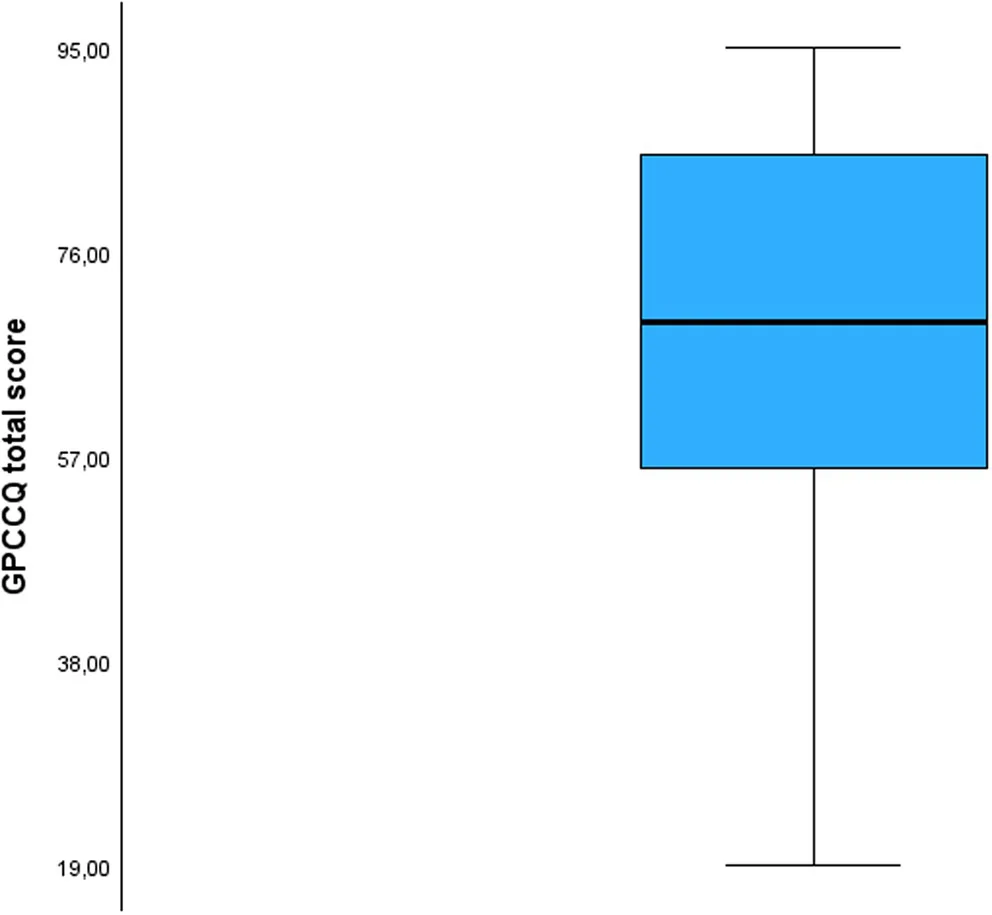
Distribution of GPCCQ total scores.

Survey responses to the contextual background questions (Table 2) showed that 89% of the participants with valid responses wished to participate in their care. Perceptions of safety were generally positive, with 71% reporting that they felt safe on the ward (BQ2) and 73% that they were satisfied with security (BQ3). However, fewer patients reported being able to discuss difficult topics without fear of reprisal (53%, BQ4) or trusting staff (59%, BQ5). These percentages represent the proportion of valid responses coded as ‘Yes’ to each question.

**TABLE 2:** Distribution of responses to contextual background questions.

Contextual background question	Yes (*n*, %)	No (*n*, %)	Partly (*n*, %)	Missing (*n*, %)
**BQ1**. Do you want to participate in the planning of your care?	286 (89)	16 (5)	20 (6)	7 (2)
**BQ2**. Do you feel safe in the ward?	229 (71)	35 (11)	57 (18)	8 (2)
**BQ3**. Are you satisfied with the security at the ward?	236 (73)	35 (11)	53 (16)	5 (2)
**BQ4**. Do you experience that you can talk about everything, even hard things, without risking punishment?	170 (53)	84 (26)	67 (21)	8 (2)
**BQ5**. Do you experience that you can trust the staff?	186 (59)	48 (15)	84 (26)	11 (3)

*Note:* Percentages for *Yes*, *No* and *Partly* are calculated from valid responses for each item. Missing percentages are calculated from the total sample (*N* = 329).

### 3.2 Examining Differences Across Background Characteristics

To explore whether overall PCC perceptions varied across background characteristics, GPCCQ total scores were compared across groups. No significant differences in total scores were found according to gender, age, ethnicity, diagnosis, or hospital type. However, Welch's one‐way ANOVA showed a statistically significant difference across length‐of‐stay groups (Welch's *F*[3, 120.81] = 3.39, *p* = 0.020, η^2^ = 0.035), with a small effect size. Games–Howell post hoc comparisons indicated a difference between the 2–5‐year and > 10‐year groups before correction (*p* = 0.041); however, this difference did not remain statistically significant after Bonferroni–Holm correction (Figure 2). When GPCCQ total scores were categorised as low, medium and high, chi‐square analyses likewise showed an association with length of stay (χ^2^[6] = 14.45, *p* = 0.025, Cramer's *V* = 0.155), while no statistically significant associations were found for the other background characteristics (Table S2). Analyses of the contextual background questions across length‐of‐stay groups are presented in Table S3.

**FIGURE 2:**
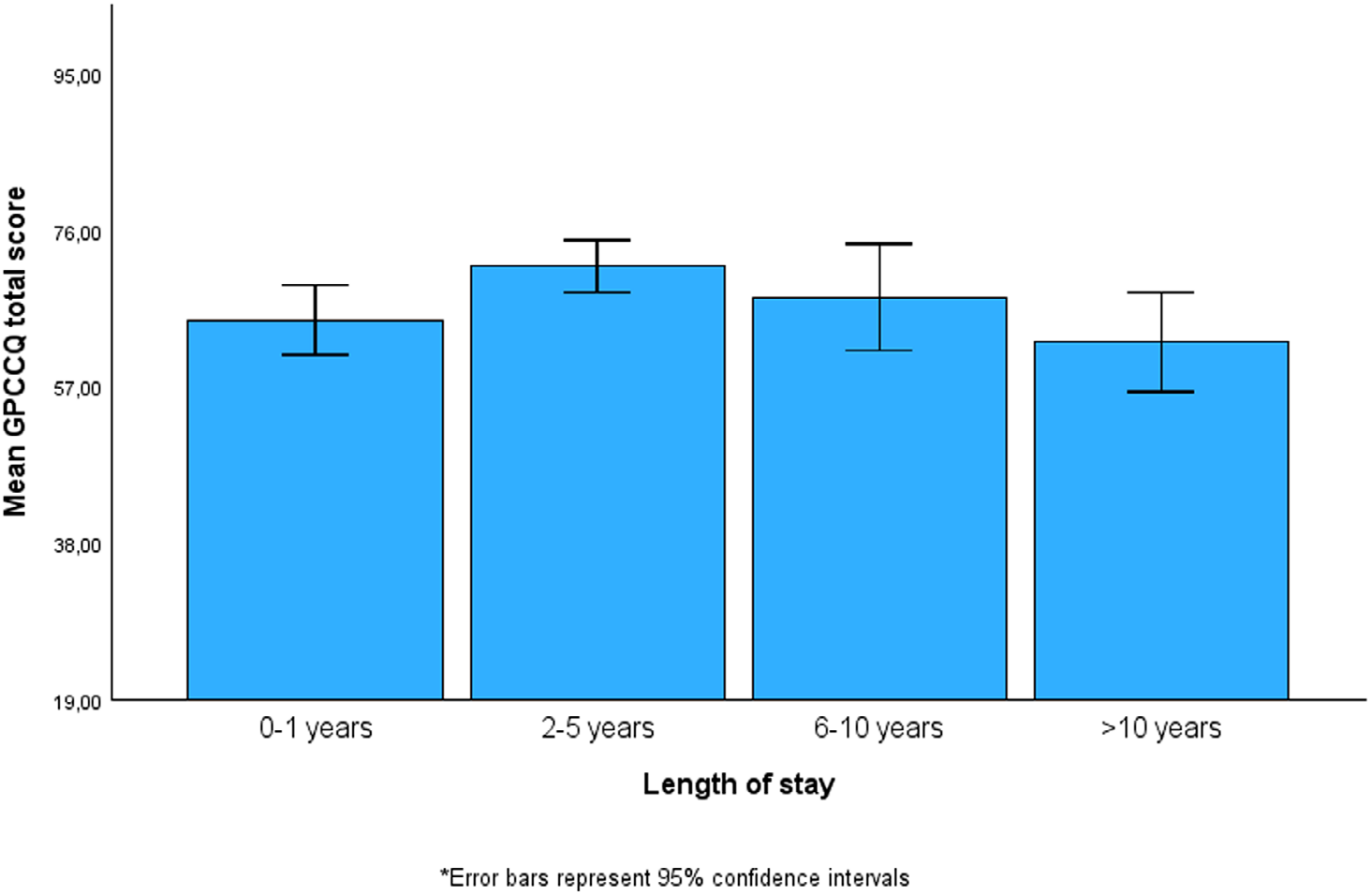
Mean GPCCQ total scores by length of stay in forensic psychiatric care*.

For the contextual background questions, significant overall differences were identified only for BQ4 and BQ5 (Table S3). For BQ4, the proportion of patients responding ‘Yes’ to the communicative‐safety question differed across length‐of‐stay groups (Welch's F[3, 138.07] = 2.75, *p* = 0.045, η^2^ = 0.026). The proportion responding ‘Yes’ decreased from 64% among patients hospitalised for 0–1 year to 41% among those hospitalised for > 10 years (Figure 3). Games–Howell post hoc comparisons indicated a difference between these groups before correction (*p* = 0.042); however, this difference did not remain statistically significant after Bonferroni–Holm correction (adjusted *p* = 0.252).

**FIGURE 3:**
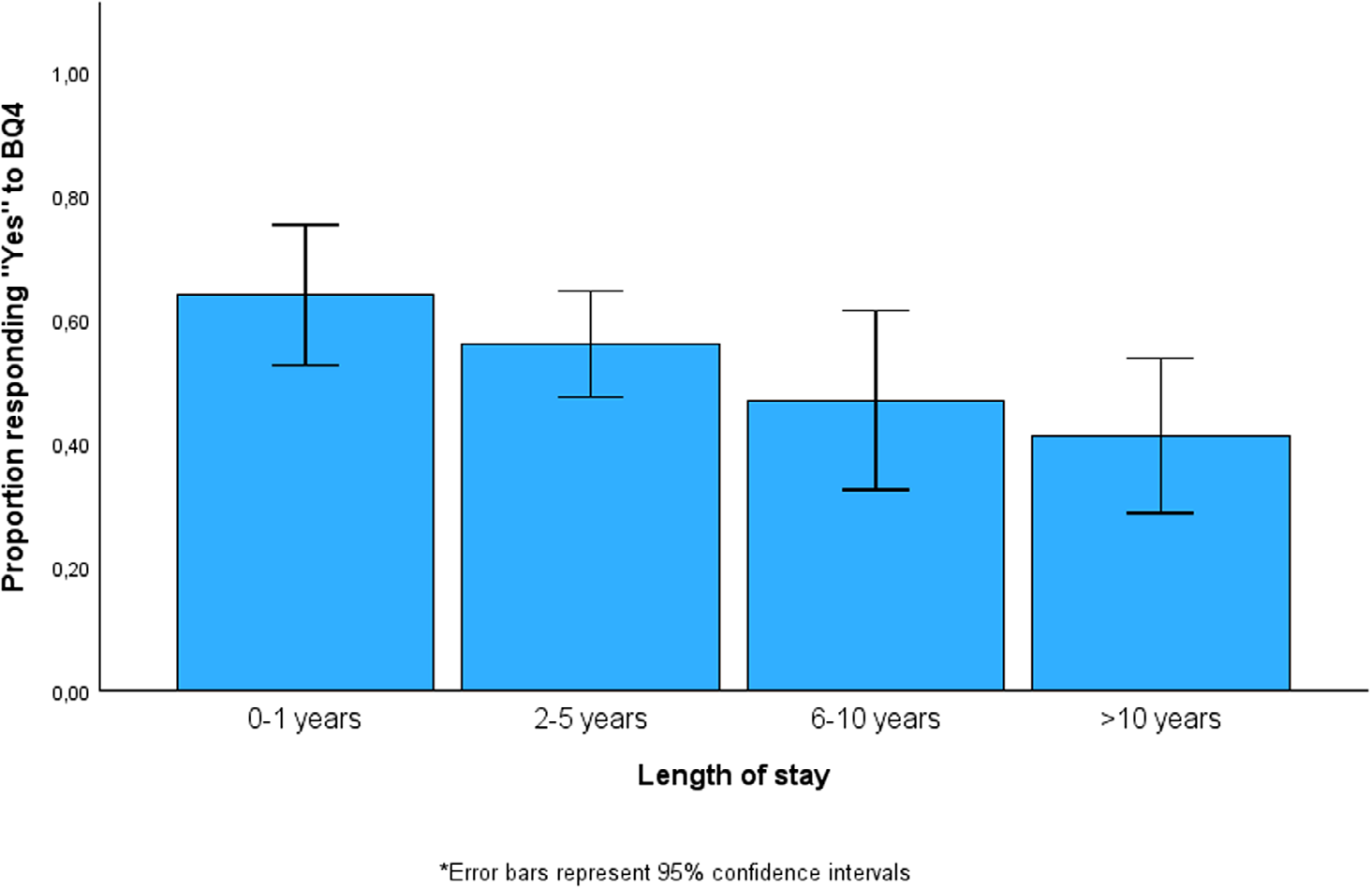
Proportion of patients responding “Yes” to communicative safety (BQ4) by length of stay in forensic psychiatric care*.

For trust in staff (BQ5), there was a statistically significant overall difference across length‐of‐stay groups (Welch's *F*[3, 134.12] = 5.92, *p* < 0.001, η^2^ = 0.056). The proportion responding ‘Yes’ was 67% among patients hospitalised for 0–1 year and 2–5 years, compared with 38% among those hospitalised for > 10 years (Figure 4). Games–Howell post hoc comparisons showed differences between the > 10‐year group and both the 0–1‐year (*p* = 0.006) and 2–5‐year groups (*p* = 0.001). Both remained statistically significant after Bonferroni–Holm correction (adjusted *p* = 0.030 and 0.006, respectively). BQ5 therefore showed the clearest association with length of stay among the contextual background questions.

**FIGURE 4:**
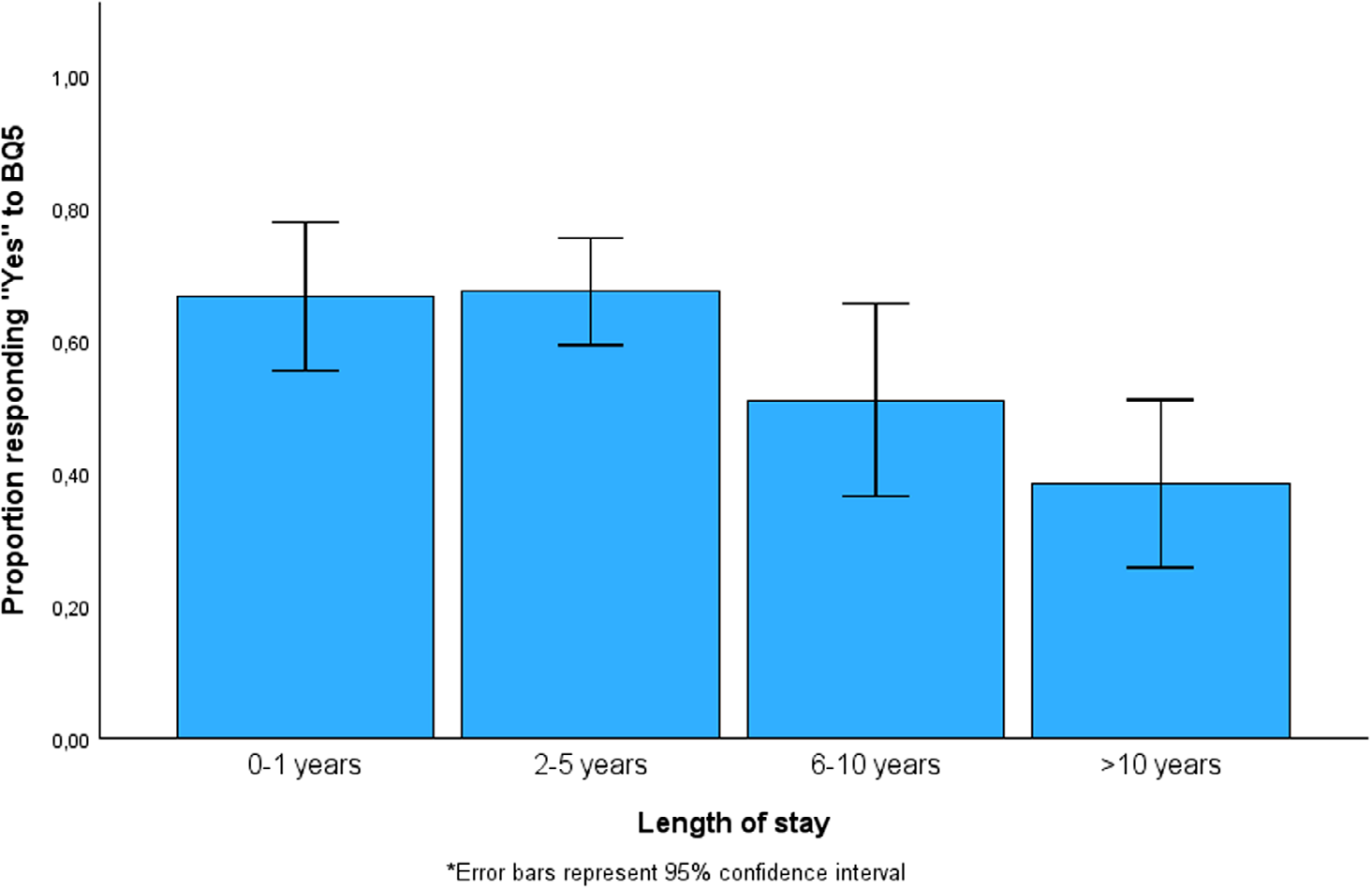
Proportion of patients responding “Yes” to trust in staff (BQ5) by length of stay in forensic psychiatric care*.

### 3.3 Identifying Areas for Improvement

To identify relative strengths and areas for improvement in PCC delivery, mean scores for the individual GPCCQ items were examined. The highest mean scores were observed for feeling respected by staff (*M* = 3.72, Q17), having the opportunity to talk to staff when feeling worried (*M* = 3.72, Q14), and receiving understandable answers to questions (*M* = 3.67, Q4). These findings indicate comparatively positive experiences of respect, communication and emotional support.

In contrast, lower mean scores were found for involvement in decisions about care and treatment (*M* = 3.10, Q7), involvement of relatives (*M* = 3.13, Q13), and use of patients' own resources in care and treatment (*M* = 3.20, Q12). These findings suggest that broader participation, shared decision‐making and involvement of relatives in care remain areas for improvement.

Of the 283 patients with a valid response to Q18, 68% reported that they and staff had developed a written care plan. Among those who reported having a written care plan and completed the follow‐up items (*n* = 186), involvement in designing the plan (*M* = 3.83, Q18a) and understanding how the plan was formulated (*M* = 4.12, Q18b) were rated relatively positively. Descriptive statistics for all GPCCQ items are presented in Table S1.

## 4 Discussion

This was the first nationwide investigation of patients' perceptions of PCC in FMHS in Sweden. Using a quantitative approach, this study provides new insights into how patients perceive the interpersonal, participatory, and structural elements of care within forensic psychiatric rehabilitation wards. The results identified areas of strength, such as respectful staff interactions, while trust and communicative safety emerged as particularly important areas for attention. These relational conditions are central to PCC, as meaningful participation and shared decision‐making depend on patients feeling able to trust staff and communicate openly.

The relatively high total scores on PCC observed in this study are consistent with recent findings from FMHS in the United Kingdom, where patients reported satisfaction scores above the mid‐point, particularly in domains related to safety, overall care and staff interactions (Khan et al. 2025). Similar to our findings, these results suggest that despite the restrictive and coercive nature of FMHS, relational and safety‐related aspects of care can be perceived positively by patients. This challenges assumptions that long‐term secure psychiatric environments are uniformly experienced as negative or poor in quality.

While relational and safety‐related aspects appeared comparatively strong, our findings indicate gaps in structural aspects of PCC, particularly involvement in decision‐making and the extent to which patients' own resources, such as their will, knowledge and capacities, were utilised in care and treatment. This suggests that interpersonal respect and relational support do not necessarily translate into active partnership and shared agency. This pattern is consistent with findings from a previous systematic review, which identified supportive care relationships as a prerequisite for participation while also highlighting structural barriers to information‐sharing, involvement and collaboration (Vidaurrazaga Aras et al. 2024). From a nursing perspective, this distinction is important. PCC extends beyond respectful interactions to recognising and incorporating patients' own knowledge, preferences and resources into care. Positive relational satisfaction should therefore not obscure limitations in opportunities for meaningful participation and co‐creation of care, particularly given the prolonged admissions common in FMHS.

### 4.1 Length of Stay, Trust and Perceived Person‐Centred Care

Length of stay was associated with variation in perceived PCC, although the clearest differences were observed in trust in staff. While continuity and familiarity have been suggested by staff as strengthening relational quality in FMHS (Sollied et al. 2023), the present findings indicate a more complex pattern, observed among patients with longer admissions and a similar, though less robust pattern for patients' ability to discuss topics without fear of reprisal. These findings align with qualitative research describing how patients in FMHS may withhold personal thoughts and opinions while adapting to institutional expectations (Humphries et al. 2023; Marklund et al. 2020). In such contexts, participation risks becoming performative rather than genuinely collaborative.

At the core of PCC lies the recognition of the individual as a unique person beyond diagnosis, offence, or risk status. Reduced trust and limited communicative safety may make this recognition of personhood difficult to sustain in clinical practice. When treatment becomes primarily diagnosis‐ or risk‐focused, it may be perceived as disconnected from patients' lived realities, leading to self‐censorship and disengagement (Green et al. 2026). Sustaining personhood within FMHS is therefore not merely a philosophical ambition, but a practical condition for meaningful participation and rehabilitative progress.

Moreover, longer hospital stays are often associated with more complex care pathways, histories of violence, and more severe or treatment‐resistant psychiatric conditions, which may further complicate therapeutic engagement and contribute to relational strain (Dima et al. 2024). While causal inferences cannot be drawn, these contextual conditions may help explain the variations observed across length‐of‐stay groups in the present findings.

### 4.2 Physical Versus Psychological Safety

Another important finding is the distinction between perceived external security (e.g., protection from harm or dangerous items) and internal psychological safety (e.g., trust and openness). Although most respondents felt physically safe on their wards, only half reported that they could speak freely without fear of reprisal. This discrepancy illustrates a paradox within FMHS: patients value a safe hospital environment as important for recovery (Katsakou et al. 2011; Valenti et al. 2014), while the structures designed to ensure safety may also constrain openness and authenticity in therapeutic relationships.

In FMHS, disagreement with staff or treatment goals may be pathologised as a symptom of illness or an indicator of risk, potentially affecting patients' progress toward discharge (Marklund et al. 2020; Pedersen et al. 2025; Vidaurrazaga Aras et al. 2024). Although rarely explicit in policy, such values are deeply embedded in daily practices and testimonies (Chappell and Jeppsson 2023; Pedersen et al. 2025).

Such dynamics may create invisible barriers to PCC. Opportunities for PCC may exist, but patients may feel compelled to align with institutional logic rather than express their own genuine goals or values (Pedersen et al. 2025; Marklund et al. 2020; Holley et al. 2020). The contrast between relatively positive ratings of physical safety and lower ratings of psychological safety can be understood in this light. Advancing PCC in FMHS therefore requires attention to the relational and cultural conditions that shape everyday interactions.

### 4.3 Variation Across Patient and Hospital Characteristics

The study did not identify significant differences in GPCCQ total scores across other background characteristics other than length of stay. The observed differences were therefore not clearly confined to the patient or hospital categories examined and may indicate broader, system‐level challenges in the delivery of PCC across FMHS. In Sweden, regional forensic psychiatric hospitals typically have a designated role in admitting patients with complex needs and higher levels of risk. Given this mandate, these settings might be expected to differ in their ability to deliver PCC, either positively through greater resources or negatively because of heightened restrictions. However, the absence of significant differences by hospital type does not establish that these challenges are systemic. Further research using more detailed organisational measures and longitudinal designs is needed to clarify whether such patterns reflect system‐level processes. At the same time, the associations observed with length of stay may reflect processes related to prolonged institutionalisation, as previous research has described how long‐term forensic psychiatric care can be accompanied by reduced autonomy, internalised surveillance and diminished agency (Pedersen et al. 2025; Chow and Priebe 2013). Taken together, these findings highlight the importance of addressing potential institutional barriers to participation and agency, particularly among patients with prolonged admissions, while further research is needed to determine the extent to which these challenges operate at a system level.

### 4.4 Strengthening Agency and Involvement of Relatives

A breakdown of the GPCCQ item‐level responses revealed a more nuanced pattern of participation. Many patients rated core relational aspects of PCC, such as respectful and supportive staff interactions, positively. In contrast, lower ratings were observed for broader involvement in decisions about care and treatment, the extent to which patients' own resources were utilised, and the involvement of relatives. The limited involvement of relatives is particularly concerning given that family contact can be a protective factor against violent behaviour in forensic psychiatric populations (de Vries Robbé et al. 2011; Völlm et al. 2018). Furthermore, the lower ratings for involvement in decision‐making are consistent with those of Valenti et al. (2014), who reported that 92% of patients receiving involuntary care felt excluded from treatment decisions. Similarly, a recent systematic literature review also highlighted gaps in the structural conditions needed to support participation in FMHS (Vidaurrazaga Aras et al. 2024).

From a nursing perspective, actively involving patients and, where appropriate, their families in care planning is a core professional responsibility. These gaps warrant particular attention given the prolonged length of stay common in FMHS and the recurring expressions of vulnerability and helplessness described in qualitative studies (Humphries et al. 2023). Developing communication strategies, structured dialogues, and approaches that include significant others in the nursing process may strengthen patients' involvement, agency, and sense of partnership in care. Moreover, this gap stands in contrast to recent legal reforms in Sweden, which mandate that all patients, including those receiving coercive care, must have their individual strengths, resources, preferences, and capacities recognised and incorporated into care planning (National Board of Health and Welfare 2022). Bridging the gap between policy and practice may require clearer implementation strategies, routine evaluation of participatory processes, and accountability mechanisms to ensure that involvement is substantive rather than symbolic.

## 5 Strengths and Limitations

The strengths of this study include its national scope, relatively large sample size, and use of a validated, person‐centred measurement instrument. However, the cross‐sectional design does not allow conclusions about the direction or causality of the associations observed with length of stay. The response rate was moderate (42%); although this is common in this population (Pedersen et al. 2021), it may introduce response bias if those who declined participation differ systematically in ways related to perceptions of PCC from those who responded.

Notably, the substantial variation in participation rates across hospitals suggests a lack of consistent infrastructure and support for research within FMHS. As highlighted by Pedersen et al. (2021), variability in recruitment practices is often associated with organisational culture, staff gatekeeping and competing clinical priorities, which can inadvertently limit access to certain patient groups—voices that may be particularly important for understanding relational and participatory barriers to PCC. This variability may hinder equitable participation in research. Finally, although robust, the survey design may have limited the depth of insight into the lived experiences and the complex relational dynamics inherent in PCC.

## 6 Conclusions and Clinical Implications

Although patients in FMHS reported positive experiences of respect and emotional support, their lower ratings of involvement in decision‐making suggest a continued gap between PCC principles and their realisation in FMHS. The absence of significant differences by hospital type suggests that these challenges were not clearly confined to one organisational category, although more nuanced differences between settings cannot be excluded.

These findings highlight an important role for forensic psychiatric nursing in developing and sustaining care models capable of maintaining trust, agency, and collaborative engagement throughout the care trajectory, including prolonged periods of hospitalisation. Such approaches may enhance patient experiences while supporting the core objectives of forensic psychiatry: reducing risk, enabling recovery, and supporting safe reintegration into society. The challenge is therefore to determine how nursing practices, organisational structures and policies can be aligned to support meaningful PCC within restrictive care contexts.

## 7 Relevance for Clinical Practice

Forensic psychiatric nursing plays a pivotal role in bridging the gap between the ideals of PCC and the restrictive clinical realities. These findings highlight the need for nurses to actively promote patient participation in care planning, foster therapeutic alliances and safeguard psychological safety, including under coercive care. Strengthening relational competencies and embedding structured strategies for trust and dialogue may support patient agency and contribute to recovery and ethical, person‐centred practice in FMHS.

## Author Contributions

V.V.A. conceived and designed the study, coordinated data collection, performed data entry, statistical analyses and wrote the majority of the manuscript. S.O. contributed to study design, data analysis, interpretation of findings and manuscript writing. E.A., A.W. and T.N. contributed to study design, guided and supported data analysis and critically reviewed and revised the manuscript. All authors approved the final version of the manuscript.

## Funding

The authors have nothing to report.

## Ethics Statement

This study was approved by the Swedish Ethical Review Authority (reference number [2022‐02148‐01]).

## Conflicts of Interest

The authors declare no conflicts of interest.

## Supporting information


**Table S1:** Descriptive statistics for individual GPCCQ items.

**Table S2:** Associations between background characteristics and perceived person‐centred care.

**Table S3:** Differences in contextual background questions across length‐of‐stay groups.

## Data Availability

The data that support the findings of this study are available on request from the corresponding author. The data are not publicly available due to privacy or ethical restrictions.
