# Supplementary material for: Time, Trust and Participation: A Nationwide Quantitative Study of Person‐Centred Care in Forensic Mental Health Settings
Source: Int J Ment Health Nurs. 2026 Oct 27;35(6):e70383. doi: 10.1111/inm.70383 (PMC13714552; doi:10.1111/inm.70383)
Supplement: Supplementary file 1 [file INM-35-0-s001.docx]

**Supplementary Table S3**

*Differences in contextual background questions across length-of-stay groups*

| **Background question** | **Welch’s F** | **df** | ***p*** | **η²** |
| --- | --- | --- | --- | --- |
| BQ1 Willingness to participate in care | 0.27 | 3, 137.04 | .846 | .003 |
| BQ2 Feeling safe on ward | 0.55 | 3, 135.18 | .650 | .005 |
| BQ3 Satisfied with security | 1.44 | 3, 138.58 | .235 | .015 |
| BQ4 Communicative safety | 2.75 | 3, 138.07 | **.045** | .026 |
| BQ5 Trust in staff | 5.92 | 3, 134.12 | **<.001** | .056 |
