# Supplementary material for: Time, Trust and Participation: A Nationwide Quantitative Study of Person‐Centred Care in Forensic Mental Health Settings
Source: Int J Ment Health Nurs. 2026 Oct 27;35(6):e70383. doi: 10.1111/inm.70383 (PMC13714552; doi:10.1111/inm.70383)
Supplement: Supplementary file 2 [file INM-35-0-s002.docx]

**Supplementary** **Table S1**

*Descriptive statistics for individual GPCCQ items*

| **Item** | **n** | **Positive %** | **Neutral %** | **Negative %** | **Mean** | **SD** |
| --- | --- | --- | --- | --- | --- | --- |
| Q1. Did the staff listen to how you experienced your health condition/illness? | 318 | 58 | 24 | 18 | 3.57 | 1.43 |
| Q2. Did you and the staff discuss how your health condition/illness can affect your daily life? | 314 | 49 | 26 | 26 | **3.24** | 1.51 |
| Q3. Were you encouraged to ask questions as you wished? | 315 | 51 | 23 | 26 | 3.28 | 1.54 |
| Q4. If you asked questions to the staff, did you receive answers that you understood? | 321 | 62 | 21 | 17 | **3.67** | 1.32 |
| Q5. Did you receive enough information about your care and treatment? | 321 | 53 | 24 | 23 | 3.48 | 1.46 |
| Q6. Did you and staff agree on next steps in your care and treatment? | 324 | 59 | 23 | 18 | 3.62 | 1.39 |
| Q7. Were you involved in making decisions about your care and treatment as much as you wished? | 322 | 42 | 34 | 24 | **3.10** | 1.49 |
| Q8. Do you feel that what is important to you in your daily life was taken into account in the planning of your care and treatment? | 315 | 51 | 25 | 23 | 3.29 | 1.49 |
| Q9. Did you and the staff formulate goals that are important to you in planning for your continued care and treatment? | 318 | 56 | 22 | 22 | 3.47 | 1.45 |
| Q10. Do you feel that the staff coordinated your contacts with other units within healthcare as much as you wished? | 311 | 53 | 26 | 21 | 3.23 | 1.58 |
| Q11. Did you and the staff discuss what you can do yourself to improve your health condition/illness? | 316 | 54 | 25 | 21 | 3.37 | 1.54 |
| Q12. Do you feel that your resources such as will, knowledge, and physical capacity were utilized in your care and treatment? | 318 | 48 | 30 | 22 | **3.20** | 1.53 |
| Q13. Were your relatives given the opportunity to be involved in your care and treatment as much as you wished? | 298 | 53 | 28 | 20 | **3.13** | 1.71 |
| Q14. Did you have the opportunity to talk to the staff if you felt worried about your health condition/illness or your care and treatment? | 320 | 64 | 19 | 17 | **3.72** | 1.41 |
| Q15. Did you feel like an equal person in the meeting with the staff? | 321 | 54 | 24 | 22 | 3.43 | 1.40 |
| Q16. Were you treated in a way that you trusted the staff? | 320 | 59 | 23 | 18 | 3.58 | 1.42 |
| Q17. Did you feel respected as a person by staff? | 324 | 62 | 19 | 19 | **3.72** | 1.37 |
| Q18 Did you and the staff together make a written plan for your continued care and treatment? | 283 | 68 | – | 32 | – | – |
| Q18a. Did you feel involved in the design of the plan for your continued care and treatment as much as you wished? | 186 | 63 | 24 | 12 | 3.83 | 1.18 |
| Q18b. Was the plan for your continued care and treatment formulated in a way that you understand it? | 186 | 76 | 16 | 9 | 4.12 | 1.11 |

*Percentages are based on valid responses. “Not applicable” responses were treated as missing. Q18 is a binary filter item. Positive responses represent scores 4–5, neutral responses score 3, and negative responses scores 1–2.
