# Supplementary material for: Time, Trust and Participation: A Nationwide Quantitative Study of Person‐Centred Care in Forensic Mental Health Settings
Source: Int J Ment Health Nurs. 2026 Oct 27;35(6):e70383. doi: 10.1111/inm.70383 (PMC13714552; doi:10.1111/inm.70383)
Supplement: Supplementary file 3 [file INM-35-0-s003.docx]

**Supplementary Table S2**

*Associations between background characteristics and perceived person-centred care*

| **Background variable** | **Statistical test** | **Test statistic (df)** | **p value** | **Effect size** |
| --- | --- | --- | --- | --- |
| Gender | Welch’s independent-samples t-test | *t*(57.219) = −0.19 | .851 | Cohen’s *d* = −0.03 |
| Age | Welch’s one-way ANOVA | F(3, 19.80) = 2.01 | .145 | η² = .014 |
| Ethnicity | Welch’s independent-samples t-test | *t*(134.52) = −0.96 | .341 | Cohen’s *d* = −0.13 |
| Length of stay | Welch’s one-way ANOVA | F(3, 120.81) = 3.39 | **.020** | η² = .035 |
| Diagnosis | Welch’s one-way ANOVA | F(6, 15.29) = 1.25 | .336 | η² = .021 |
| Hospital type | Welch’s independent-samples t-test | *t*(287.28) = 0.90 | .370 | Cohen’s *d* = −0.10 |

**Panel B.** *GPCCQ low/medium/high categories*

| **Background variable** | **Statistical test** | **Test statistic (df)** | **p value** | **Effect size** |
| --- | --- | --- | --- | --- |
| Gender | Pearson’s chi-square | χ²(4) = 5.09 | .278 | Cramer’s V = .092 |
| Age | Pearson’s chi-square | χ²(6) = 12.59 | .050 | Cramer’s V = .145 |
| Ethnicity | Pearson’s chi-square | χ²(2) = 0.78 | .677 | Cramer’s V = .051 |
| Length of stay | Pearson’s chi-square | χ²(6) = 14.45 | **.025** | Cramer’s V = .155 |
| Diagnosis | Pearson’s chi-square | χ²(12) = 7.32 | .836 | Cramer’s V = .112 |
| Hospital type | Pearson’s chi-square | χ²(2) = 1.51 | .470 | Cramer’s V = .071 |
